# Post-Stroke Depression: Main Phenomenological Clusters and their Relationships with Clinical Measures

**DOI:** 10.3233/BEN-2012-110236

**Published:** 2012-02-20

**Authors:** Davide Quaranta, Camillo Marra, Guido Gainotti

**Affiliations:** Research Center for NeuropsychologyDepartment of NeuroscienceCatholic UniversityRomeItaly

**Keywords:** Post-stroke depression, Post stroke depression Rating Scale, factor analysis

## Abstract

*Objectives:* To investigate the principal psychopathological dimensions of post-stroke depression (PSD) through the assessment of the factorial structure of the Post-Stroke Depression Rating Scale (PSDRS).

*Methods:* We enrolled ninety-eight subjects with PSD, who underwent the PSDRS, MMSE and Barthel Index. Information about demographic, clinical, and neuroanatomical factors was collected.

*Results:* The factor analysis extracted three factors accounting for 63.4% of the total variance, and identified as: (1) “Depressive and Anxious Symptoms“ (DAS); (2) “Lack of Emotional Control” (LEC); 3) “Reduced Motivation” (RM). On multivariate statistics, DAS severity was predicted by previous history of mood disorders and Barthel Index; LEC severity was predicted by Barthel Index; RM severity was predicted by age.

*Conclusions:* The PSDRS displayed a reliable factor structure that agreed with previous interpretation of PSD. In particular, core depressive symptoms seem to be related to premorbid personality and functional status, whereas apathy/anhedonia may be connected to brain aging.

## 1. Introduction

Post-stroke depression (PSD) is the most common behavioral consequence of stroke, affecting about 40% of patients [19,30], and influencing rehabilitative outcome [13], quality of life [22], cognition [23], and survival [47].

Notwithstanding the clinical relevance of PSD has become evident, the mechanisms underlying its occurrence and phenomenological features have not been clarified yet.

The theoretical debate on PSD has been mainly, even if not exclusively, focused on its possible neuroanatomical substrate. Several studies have reported an association between PSD and specific site of lesion. In most positive studies, PSD was dichotomized into two distinct forms: major PSD and minor PSD, resembling the correspondent forms of primary depression identified by DSM. Major PSD has been frequently associated to left frontal lesions [37,45,46]; furthermore, a modification of such anatomical substrate during the post-stroke period has been proposed [40]. Data from Hama et al. [20] have suggested differential anatomical substrates of two different subtypes of PSD: “apathetic” PSD, associated to bilateral basal ganglia damage, and “affective” PSD, associated to left frontal lobe lesions.

This approach has been widely criticized, mainly because of the controversies in assimilating PSD to endogenous mood disorders. In particular, DSM-III and DSM-IV criteria for mood disorders have been often used to diagnose PSD [36]; this approach may have been misleading because some symptoms considered as diagnostic of depression (such as lack of concentration, and sleep and appetite disorders) can frequently occur also in non-depressed stroke patients. It must be acknowledged, however, that, making reference to

ISSN 0953-4180/12/$27.50 © 2012 – IOS Press and the authors. All rights reserved studies conducted by Federoff et al. [11] and Paradiso et al. [31] Robinson [36] concluded that modifying DSM-IV criteria because of the existence of these symptoms is probably unnecessary.

When a specific assessment scale (the Post-Stroke Depression Rating Scale, PSDRS) was used, clinical profile of "major" and "minor" PSD overlapped, and they were clearly distinguishable from the one of primary Major Depressive Disorder (MDD) [15]. Furthermore, several studies were not able to replicate the association between PSD and left frontal lesions [14, 33] or identified different anatomical substrates [27]. A comprehensive meta-analysis conducted on this topic [10] did not support any possible association between PSD and lesion site.

An alternative, psychological interpretation of PSD has been suggested, proposing that the personal salience of functional deficits following stroke may be one of the main determinants of PSD onset [16]. As a matter of fact, the relationship between PSD and functional impairment is complex, and several variables could determine the occurrence of PSD.

Several authors reported a clear association between scores reported at clinical scales for autonomy and PSD [23,33,46] but other studies did not replicate these findings [24,27]. However, standard scales for the evaluation of functional status may provide insufficient information about the subjective consequence of neurological deficits on each post-stroke depressed subject. In this sense, an interpretation based on the attribution of a personal meaning to neurological deficits may be more reliable to explain the onset of PSD [16].

Further evidence supporting the role of psychosocial factors in PSD came from psychological studies, that stressed the role of major life events [9] and of cognitive factors, such as recovery locus of control [44], on the development of PSD.

Besides the methodological limitations connected to the diagnosis PSD and the controversies about its anatomical substrate, other factors, such as heterogeneity of study sets (hospitalized subjects, patients in rehabilitative structures, or outpatients); differences in time from stroke onset; unavailability of reliable diagnostic tools for PSD [7] can account for the contradictions existing among previous studies. In fact, there is a lack of assessment scales specifically devised for PSD. In last years several psychiatric scales have been applied to PSD, and some of them have received diagnostic validation [1,6]. Nevertheless, they suffer from the limitation of having been developed for patients with endogenous depression. To address this problem, the Post-Stroke Depression Rating Scale (PSDRS) has been recently validated for diagnostic purposes [35].

The availability of a more specific assessment scale could allow a more accurate investigation of clinical correlates of PSD.

In previous studies, apart from the disputable distinction between its "major" and "minor" forms, PSD has been mostly considered as a homogeneous entity. Clinical and anatomical correlates have been evaluated as risk factors or severity modulators of this rather unitary construct. Nevertheless, it is possible that the clinical presentation of PSD may be the result of different interacting "vectors", that, in turn, may be caused or modulated by different factors.

The present work was aimed to investigate the factorial structure of the PSDRS in subjects affected by PSD, attempting to identify the main phenomenological clusters of PSD and their clinical correlates.

## 2. Materials and methods

One-hundred and forty-three (143) inpatients of the Neurorehabilitation Unit of our institution were consecutively screened for PSD. All of them were affected by ischemic stroke that occurred less than 6 months prior to evaluation. Patients were enrolled in the study if they fulfilled DSM-IV-TR criteria for mood disorder due to stroke, with depressive manifestations [3], that is the currently accepted diagnostic codification of PSD.

Exclusion criteria were: previous stroke; other neurological diseases, in particular neurodegenerative conditions potentially associated to depressive manifestations; severe acute or chronic medical illness; aphasia severe enough to prevent from a reliable clinical evaluation of affective conditions; severe cognitive impairment (Mini Mental State Examination [MMSE] $< 10$). No limitations about the functional status were set for the enrollment.

Cognitive status was globally evaluated using MMSE, whereas functional level of autonomy was quantified by means of the Barthel Index (BI).

The psychiatric history of each patient was assessed using a semi-structured interview comprising three questions regarding: psychiatric diagnosis; previous admissions in psychiatric ward; use of psychotropic medications. If the answer to one of these questions was positive, more detailed information was collected; furthermore, data about psychiatric history should be confirmed by a family member to be taken into account. Subjects with a previous history of psychiatric disorders other than mood or anxiety disorders were excluded from the study. Data about familiarity for psychiatric disorders were also collected. The presence of recent stressful events (RSE) during the six months prior to stroke (according to the timing adopted by Bush [9]) was assessed. Demographic data about marital status and income (< 10.000 euro/year = low income; 10.000–35.000 euro/y = medium income; > 35.000 euro/year = high income) were collected.

The anatomical site of lesions was determined by an expert stroke neurologist (CM) who examined neuroradiological findings (CT or MRI) and was blind to the results of the psychiatric examination of each patient; lesions were classified as anterior or posterior by determining the position of their center in respect to the central sulcus. Furthermore, strokes were also considered to have a subcortical extension if basal ganglia, internal capsula, thalamus, or deep white matter were interested.

All the enrolled patients underwent the Post-Stroke Depression Rating Scale [15], which was administered by a trained neurologist in the late morning or in the early afternoon.

The PSDRS consists of ten sections exploring: depressed mood; guilt feelings; death/suicidiary ideation; vegetative symptoms (i.e., appetite and sleep disorders); apathy; anxiety; catastrophic reaction; hyperemotionalism; anhedonia; diurnal variations. The score of the first nine sections is assigned on a likert scale ranging 1–5, with higher scores for more severe symptoms. The tenth section investigates diurnal mood variations that could be motivated, i.e. connected to the occurrence of frustrating events, stressing handicaps and disabilities, or unmotivated, i.e. prevalent in the early morning as is usually the case in MDD. This section is, therefore, a descriptive one which gives information about the motivated or unmotivated nature of PSD, but not about its severity. The total PSDRS score, used for screening and diagnostic purposes, is obtained by the sum of Sections 1 to 9.

## 3. Statistical analysis

In order to explore the presence of main phenomenological clusters of PSD, identifiable by the PSDRS, a factor analysis on sections 1–9 was conducted. Only factors with eigenvalue > 1 were taken into account, after a confirmatory visual inspection of the scree plot. Sample adequacy was calculate by Kayser-Meyer-Olkin (KMO) method. The matrix was rotated using promax method, a non-orthogonal technique, with Kaiser normalization. A regression method was used to attribute each subject a factor score on the basis of the scores reported in predicting variables. Loadings > 0.500 were considered as significant in the definition of each factor.

The relationship between continuous variables was evaluated by determining Pearson's r; group comparison were conducted using t-test for independent sample (with Levene's test for equality of variance) or one-way ANOVA followed by post-hoc Tukey's test as appropriate.

Finally, the identified factors were set as the dependent variables of linear regression models in which variables showing a significance level of $p \leqslant 0.20$ at univariate analyses were set as possible predictors. Regression models were obtained by using a backward stepwise method.

All statistics were carried out by SPSS software (version 15).

## 4. Results

A cohort of ninety-eight subjects (45 women) was enrolled in the study. Thirteen subjects (9% of the screened population) were excluded for severe aphasia; seven subjects (5%) were affected by severe chronic or acute medical illness; nine subjects (6%) were affected by a previous stroke; eight (6%) subjects were affected by other neurological disorders; three subjects (2%) scored 10 or less on MMSE; finally, five subjects (3%) displayed a combination of at least two of the above mentioned conditions.

Mean age of the selected sample was 63.00 years (SD = 11.20), with mean education of 8.44 school years (SD = 5.02); mean BI was 11.17 (SD = 6.03), and mean MMSE score was 23.88 (SD = 5.02). Marital status was distributed as follows: 10 never-married, 66 married; 4 divorced; 18 widows; in order to make data more interpretable, subjects were stratified into two categories: "currently married" (N = 66) and "currently non-married" (N = 32). Thirty-five patients had low income, 48 had medium income and 15 had high income. Patients were assessed at a mean latency stroke onset of 3.27 months (SD = 2.83); mean PSDRS total score was 16.31 (SD: 6.46).

The study sample resulted to be adequate to perform a factor analysis of PSDRS (KMO measure of sample adequacy = 0.674; Bartlett's test of sphericity: $\chi^2 = 232.9$; $p < 0.001$). On the basis of Cattell's scree test

Table 1
Factor loadings of PSDRS items after promax rotation

| | Factor I (eigenvalue: 3.04) | Factor II (eigenvalue: 1.58) | Factor III (eigenvalue: 1.12) | Communality |
|---|---|---|---|---|
| Depression | **0.562** | 0.382 | −0.069 | 0.583 |
| Guilt feelings | 0.384 | 0.169 | 0.128 | 0.271 |
| Suicide | **0.752** | −0.126 | −0.207 | 0.471 |
| Vegetative symptoms | **0.718** | −0.274 | 0.335 | 0.696 |
| Apathy | −0.095 | 0.155 | **0.872** | 0.761 |
| Anxiety | **0.707** | 0.345 | −0.057 | 0.655 |
| Catastrophic reaction | −0.015 | **0.801** | 0.234 | 0.725 |
| Hyperemotionalism | −0.021 | **0.889** | −0.122 | 0.773 |
| Anhedonia | −0.021 | −0.066 | **0.903** | 0.798 |
| Variance explained* | 33.8% | 17.5% | 12.4% | |
| Interpretation | Depressive/anxious symptoms | Lack of emotional control | Reduced motivation | |

*unrotated solution.

and of the observed *eigenvalues*, three factors were extracted, accounting for 63.7% of total variance. After promax rotation, a simple structure was obtained, with no complex variables (that is, variables with substantial loadings on more than one factor). Furthermore, the variables were adequately explained by this factor analysis, as communality values were quite high (Table 1).

The first factor (*eigenvalue* = 3.04; 33.8% of total variance explained) was defined by depression, suicidal attitude and/or thoughts, sleep and appetite disturbances and anxiety. We labelled this factor as "Depressive and Anxious Symptoms" (DAS) as it comprised the core features of anxiety and mood alteration, with disturbances of ideation and vegetative functions.

The second factor (*eigenvalue* = 1.56; 17.5% of total variance explained) was defined by hyperemotionalism and catastrophic reaction, and it was interpreted as the expression of "Lack of Emotional Control" (LEC) in patients affected by PSD.

The third factor (*eigenvalue* = 1.12; 12.4% of total variance explained) was identified by apathy and anhedonia, i.e. by "negative" symptoms; we labelled it as "Reduced motivation"(RM).

Correlations between factors scores and demographic and clinical variables are reported in Table 2; group comparisons of factors scores are reported in Table 3.

DAS scores were inversely correlated to BI score ($r = -0.386$; $p < 0.001$); worse scores were reported by female subjects ($t = 2.839$; $p = 0.006$) and by patients with a previous mood disorder ($t = 2.425$; $p = 0.018$).

LEC scores were only inversely correlated to BI scores ($r = 0.039$; $p = 0.039$).

RM scores were directly correlated to age at examination ($r = 0.250$; $p = 0.013$) and there was a trend to an inverse correlation with MMSE scores ($r = -0.189$; $p = 0.062$). Finally, RM scores were different among the four lesion location groups ($F = 2.740$; $p = 0.048$); patients with left anterior lesions showed the highest scores, while patients with right anterior lesions reported the lowest score; this difference reached statistical significance at post-hoc Tukey's test ($p = 0.05$).

Variables displaying a significance level $\leqslant 0.20$ were entered as possible predicting variables in three backward stepwise linear regression model in which DAS, LEC and RM individual scores were set as dependent variables. As the four groups of patients classified according to lesion location were completely independent, lesion location were set as four possibly predicting independent variables; this approach was chosen in order to evaluate the role of each single lesion group in determining the factor scores.

Age, gender, BI, MMSE score, income level; previous mood/anxiety disorders; RSE; and lesion location set as possibly predictive variables into the regression model for DAS. In the final model, DAS scores were predicted by a history of mood disorders (B = 0.554; Standard Error [SE] = 0.192; $p = 0.005$) and by BI index (B = −0.071; SE = 0.015; $p < 0.001$).

Linear regression model with LEC as dependent variable initially included only BI and MMSE scores; in the final model, only BI scores predicted LEC factor scores (B = −0.035; SE = 0.017; $p = 0.039$).

The regression model for RM initially included age; BI and MMSE scores; lesion location; familiarity for mood disorders. In the final model, higher age (B = 0.019; SE = 0.009; $p = 0.032$) and left anterior lesions (B = 0.553; SE = 0.221; $p = 0.014$) predicted higher RM scores.

## 5. Discussion

The investigation of mood disorders following stroke has been strongly influenced by the absence of specific

Table 2
Linear correlations between factors extracted from the PSDRS and demographic and clinical variables

| | DAS | | LEC | | RM | |
|---|---|---|---|---|---|---|
| | r | p | r | p | r | p |
| Age | 0.181 | 0.075 | 0.105 | 0.302 | 0.250 | **0.013** |
| Education | −0.074 | 0.471 | −0.041 | 0.687 | 0.045 | 0.659 |
| Latency from stroke | 0.053 | 0.606 | 0.089 | 0.381 | −0.051 | 0.619 |
| BI | −0.386 | < **0.001** | −0.211 | **0.039** | −0.169 | 0.100 |
| MMSE | −0.133 | 0.193 | −0.037 | 0.179 | −0.189 | 0.062 |

DAS: Depressive and anxious symptoms; LEC: lack of emotional control; RM: reduced motivation. Numbers in bold indicate statistical significance ($p < 0.05$); numbers in italics indicate significance level that allowed variables to enter the linear regression model ($p \leqslant 0.20$).

Table 3
Group comparisons of individual factors scores

| | DAS | | LEC | | RM | |
|---|---|---|---|---|---|---|
| **Demographic variables** | | | | | | |
| Gender | | | | | | |
| Female | 0.30 (0.975) | **t = −2.839** | 0.004 (1.067) | $t = 0.034$ | 0.13 (1,045) | $t = 1.234$ |
| Male | −0.25 (0.114) | **p = 0.006** | −0.003 (0.950) | $p = 0.973$ | −0.11 (0.955) | $p = 0.220$ |
| Marital status | | | | | | |
| Married | −0.04 (0.953) | $t = 0.508$ | 0.03 (0.934) | $t = -0.378$ | −0.03 (1.019) | $t = 0.450$ |
| Not-married | 0.07 (1.103) | $p = 0.612$ | −0.05 (1.138) | $p = 0.706$ | 0.07 (0.973) | $p = 0.654$ |
| Income level | | | | | | |
| Low | 0.25 (0.801) | *F = 2.221* | −0.05 (1.065) | $F = 1.084$ | 0.18 (1.007) | $F = 1.135$ |
| Medium | −0.21 (1.070) | *p = 0.114* | 0.13 (0.976) | $p = 0.342$ | −0.15 (0.969) | $p = 0.326$ |
| High | 0.08 (1.102) | | −0.29 (0.906) | | 0.04 (1.072) | |
| **Psychiatric history** | | | | | | |
| Previous depressive/anxious disorders | | | | | | |
| Yes | 0.31 (1.006) | **t = −2.415** | 0.004 (0.975) | $t = 0.031$ | 0.12 (0.969) | *t = −0.914* |
| No | −0.18 (0.958) | **p = 0.018** | −0.002 (1.022) | $p = 0.975$ | −0.07 (1.019) | *p = 0.363* |
| Familiar history of depression | | | | | | |
| Yes | 0.23 (0.827) | $t = -0.913$ | −0.08 (0.714) | $t = 0.337$ | −0.37 (0.864) | $t = 1.488$ |
| No | −0.04 (1.025) | $p = 0.363$ | 0.01 (1.043) | $p = 0.737$ | 0.06 (1.013) | $p = 0.140$ |
| Recent stressful events | | | | | | |
| Yes | 0.21 (1.035) | *t = −1.636* | 0.07 (0.973) | $t = -0.553$ | −0.05 (1.010) | $t = 0.358$ |
| No | −0.13 (0.963) | *p = 0.105* | −0.045 (1.022) | $p = 0.581$ | 0.03 (1.001) | $p = 0.721$ |
| **Lesion characteristics** | | | | | | |
| Lesion location | | | | | | |
| Left anterior | −0.12 (0.954) | *F = 1,699* | 0.05 (0.944) | $F = 0.500$ | 0.31 (1.004)* | **F = 2.740** |
| Right anterior | 0.14 (1.008) | *p = 0.173* | −0.10 (0.964) | $p = 0.683$ | −0.31 (0.928)* | **p = 0.048** |
| Left posterior | −0.48 (0.821) | | −0.13 (0.878) | | −0.16 (0.766) | |
| Right posterior | 0.23 (1.100) | | 0.22 (1.254) | | 0.23 (1.128) | |
| Subcortical extension | | | | | | |
| Yes | 0.02 (0.953) | $t = -0.459$ | −0.03 (0.994) | $t = 0.725$ | 0.05 (1.035) | $t = -1.111$ |
| No | −0.10 (1.212) | $p = 0.647$ | 0.15 (1.039) | $p = 0.470$ | −0.24 (0.812) | $p = 0.269$ |

DAS: Depressive and anxious symptoms; LEC: lack of emotional control; RM: reduced motivation.
Numbers in bold indicate statistical significance ($p < 0.05$); numbers in italics indicate significance level that allowed variables to enter the linear regression model ($p \leqslant 0.20$).
*statistically significant ($p < 0.05$) difference at post-hoc Tukey's test.

clinical assessment scales. This limitation may have determined the lack of a systematic approach to its underlying psychopathological dimensions. In this study, we report the results of a factor analysis of the PSDRS [15], an assessment scale specifically developed for PSD.

Three main factors has been identified, corresponding to three plausible phenomenological clusters characterizing PSD: depressive/anxious symptoms (DAS), lack of emotional control (LEC), and reduced motivation (RM).

DAS appear to be strongly correlated to the presence of previous mood disorders and to the autonomy degree of the subject. Previous mood disorders are a recognized risk factor for PSD [30] and even previous anxious symptoms are a risk factor for anxiety disorders after stroke [28]. According to our data, it is conceivable that, in these cases, the pre-existence of a personal predisposition toward anxiety and/or depression may contribute to specifically exacerbate these symptoms. Female subjects were characterized by higher DAS scores; this result is in agreement with data from previous studies both on PSD and primary depressive disorders [2,30].

Interestingly, higher DAS severity is also predicted by lower BI scores. This result could confirm the importance of the functional status in determining the severity of depressive and anxious manifestations, as reported in previous studies [23,33,46]. Furthermore, the correlation between Factor I and BI may support the hypothesis that both the appraisal of the functional deficits (that could lead to depressive symptoms) and the expectations about incomplete recovery (that may be a source of anxiety) may participate in the relationship between functional autonomy and mood changes.

BI is also the unique predictor of the severity of LEC; this factor included the scores obtained on the "catastrophic reaction" and "hyperemotionalism" subscales of the PSDRS. This result could be quite surprising, since the abnormalities of the emotional regulation are often seen as a direct consequence of the anatomical damage [38]. In previous studies, catastrophic reaction in post-stroke subjects has been linked to a higher frequency of anterior and subcortical lesions [43]; on the other hand, lesion studies have reported various injury sites in connection to hyperemotionalism, even in this case with a predominance of lesions of frontal and subcortical structures [24,38]. Nevertheless, a specific investigation of such phenomena in a selected PSD sample has not been conducted. In our sample the LEC factor was strongly related to BI, and not to the neuroanatomical variables taken into account; thus, it is conceivable that the clinical manifestation of a reduced emotional control, expressing as catastrophic reaction (i.e., a progressive lost of control with prominent emotive manifestation when patients are facing their difficulties) and/or uncontrolled emotional outbursts, could also derive from the perception of functional deficits. Furthermore, in our sample the emotional discharge was more often related to a distressing situation (as in most cases it consisted of outburst of crying) than usually reported in the definition of *emotionalism* or pseudobulbar affect [38]. These data lead to the hypothesis that these symptoms may represent an easily elicited loss of emotional control in patients with an underlying depression, more than a manifestation of pseudobulbar affect interpreted as a pure "liberation" phenomenon. The relation between LEC and depression in stroke patients is confirmed by the similar results obtained by Andersen [4] and by Gabaldon et al. [12] treating with the selective serotonin reuptake inhibitors citalopram and sertraline stroke patient with PSD and hyperemotionalism. According to these results, increased emotionalism of stroke patients could be considered as a facet of PSD.

The Factor III, defined as Reduced Motivation, was identified by the combination of apathy and anhedonia, confirming previous findings in stroke survivors [32].

We found a significant role of older age in determining the severity of these "negative symptoms". Previous studies have reported an association between older age and apathy in stroke survivors [8,42] and normal elderly subjects [26]. Furthermore, apathy is significantly more represented in late-onset depression than in early onset depression [25].

The association between older age and apathy has been generally interpreted as the expression of the physiological involution of dopaminergic circuitry in normal aging [5,26], or as a "consequence of response to uncontrollable stress and problems associated with ascending age" [5].

Patients with left anterior lesions reported higher scores on Factor III. Results from previous studies investigating the anatomical substrate of post-stroke apathy are quite inconsistent. It has been related to right side of lesion [8]; furthermore, it has been related to bilateral subcortical damage [20] and to reduced concentration of N-acetyl-aspartate in left frontal lobe [18], independently from the site of lesion. Finally, a functional study has reported a relationship between apathy and hypoperfusion of both frontal lobes after stroke [29]. Interestingly, Andersson et al. [5] reported that Apathy Evaluation Scale (AES) mean score was higher in right damaged patients, but that only in left damaged patients it was correlated to the severity of depressive symptoms.

However, these studies were heterogeneous in respect of typology of the study sample; definition and assessment scales of apathy; time elapsed from stroke. Furthermore, with the exception of the study by Hama et al. [20], in none of them the issue of apathy in depressed subjects have been specifically addressed.

Studies on patients affected by MDD have reported the melancholic subtype of MDD, in which negative symptoms are prominent, to be associated to structural [34] and functional [17] abnormalities of anterior regions of left hemisphere. A relationship between apathy and dysfunction of left anterior regions of the brain has been reported also in neurological and psychiatric disorders other than stroke, such as early Alzheimer's Disease [21] and schizophrenia [41].

However, our data must be interpreted cautiously, for the presence of possible methodological and theoretical pitfalls; from the methodological point of view, the anatomical classification we used was really simple, as information was derived from heterogeneous (i.e., CT and MRI) neuroradiological technique and thus it was not possible to conduct a more refined analysis; from the theoretical point of view, it must be considered that apathy and anhedonia are complex clinical phenomena, possibly characterized by different dimensions, that may be related to different cerebral structures of both hemispheres, as observed in schizophrenic subjects [39].

The finding of a reliable factor structure of the PS-DRS, that was specifically devised to assess mood disorders following stroke, could contribute to a better comprehension of the phenomenological characteristics of PSD. In our opinion, even if not definitive at all, our results could lead to an updated model for the pathogenesis of PSD, in which the clinical phenomena observed in PSD may be the expression of several (demographic, clinical and neuroanatomical) interacting factors in determining the clinical features of the single PSD patients. Moreover, it is conceivable that these data, if confirmed on independent samples, may also be helpful in therapeutic decision making and on monitoring the effect of pharmacological and rehabilitative treatment.

## Acknowledgments

The authors have no conflict of interest to declare.
